# Supplementary material for: Content-rich biological network constructed by mining PubMed abstracts
Source: BMC Bioinformatics. 2004 Oct 8;5:147. doi: 10.1186/1471-2105-5-147 (PMC528731; doi:10.1186/1471-2105-5-147)
Supplement: Additional File 2 — The original results of the above study (non-essential files are deleted to keep the file size under the limit set by BMC bioinformatics). [file 1471-2105-5-147-S2.bz2 › chilibotAdditionalFile2/dip10/426ID11470772E10356/html/CCR1_CCL1.html]

 


 **CCR1** and **CCL1** 
  
Found 34 abstracts in PubMed, retrieved 10.  
 

 What does Google say? 
 PDF only 
| .edu only 

---

**Interactive relationship** (e.g. stimulation, inhibition, etc)

**Non-interactive relationship** (e.g. studied together, co-existance, homology, etc.)

- Renal function urine volume UV, 24 h excretion of Na, K and Cl UNA V, UK V, UCl V, clearance of Na CNa, K CK and Cl CCl  [ **CCL1** ] , creatinine CCr  [ **CCR1** ] , osmotic substances Cosm, and solute free water FWC, and the fractional excretion of Na, K and Cl and ALDO were measured for an additional 3 consecutive days during the training period.  Ref: 12405745 Equine Vet J Suppl, 2002
- To test the in vivo relevance of this, we used a murine melanoma system and knockout mice to investigate the function of the chemokine receptor CCR5 and its ligands, CCR  [ **CCR1** ]  ligand CCL  [ **CCL1** ]  3 and CCL5.  Ref: 12682253 J Immunol, 2003
- CCL  [ **CCL1** ]  receptors, CCR2,  **CCR1** , and CCR5, also showed increased expression in mdx muscle.  Ref: 12609504 Neuromuscul Disord, 2003
